# Coexistence of Immune Checkpoint Inhibitor-Induced Autoimmune Diabetes and Pancreatitis

**DOI:** 10.3389/fendo.2021.620522

**Published:** 2021-04-13

**Authors:** Amy L. Zhang, Fang Wang, Lee-Shing Chang, Marie E. McDonnell, Le Min

**Affiliations:** Division of Endocrinology, Diabetes and Hypertension, Brigham and Women’s Hospital, Boston, MA, United States

**Keywords:** autoimmune diabetes, immunotherapy, pancreatitis, endocrine toxicity, immune check inhibitor (ICI)

## Abstract

Autoimmune diabetes is a rare but severe endocrine toxicity induced by immune checkpoint inhibitor (ICI) treatment. It is unclear if ICI causes selective islet toxicity or non-selective pancreas toxicity. We analyzed 11 patients treated with ICI who developed ICI-related autoimmune diabetes. Eight patients had lipase and/or amylase tested on the same day of diagnosis of autoimmune diabetes. Among them, 75% (6/8) had normal lipase and 100% (6/6) had normal amylase. There was no correlation between glucose level at onset and biochemical pancreatitis. We characterized the clinical features of ICI-induced autoimmune diabetes. Fifty-five percent (6/11) of patients tested positive for GAD65 autoantibodies, and 55% (6/11) developed diabetic ketoacidosis at manifestation of hyperglycemia. In all 11 patients, C-peptide levels were low in the presence of hyperglycemia. ICI-induced thyroiditis was found in 64% (7/11), of which 36% (4/11) were newly diagnosed with thyroiditis while the remaining 27% (3/11) had pre-existing hypothyroidism followed by ICI-induced thyroiditis. Additionally, 27% (3/11), developed ICI-induced hypophysitis. Thyroiditis and autoimmune diabetes coexisted in all patients with ICI-induced hypophysitis. The median time from ICI treatment to the onset of autoimmune diabetes was 11 weeks. Our data suggest that few patients had coexistent ICI-induced autoimmune diabetes and pancreatitis, suggesting ICI mainly caused selective islet toxicity.

## Introduction

Unleashing the immune system by using immune checkpoint inhibitors (ICIs) can improve overall survival for patients with advanced cancers (1). Since the FDA approval of ipilimumab (human IgG1 k anti-CTLA-4 monoclonal antibody) in 2011, six more ICIs have been approved for cancer therapy (2).

Despite the often durable clinical benefits of the immune checkpoint blockade therapy, ICI use is associated with a spectrum of adverse effects related to the mechanism of action, commonly referred to as immune-related adverse events (irAEs). Autoimmune diabetes induced by ICIs is a rare irAE occurring in less than 1% of patients treated with ICIs (3), but it can be life-threatening. Studies of autoimmune diabetes thus far have been limited to case reports or case series, with variable clinical presentations. Patients who develop ICI-induced autoimmune diabetes are older than those presenting with classic type 1 diabetes, often require admission for treatment, and require insulin treatment (4–10). ICI-induced autoimmune diabetes is more commonly associated with anti-PD-1 monotherapy or combination anti-PD-1 and anti-CTLA-4 therapy but has been associated with anti-CTLA-4 or anti-PD-L1 monotherapy, albeit less commonly (11). Anti-GAD65 positivity, HLA type DR4, and preexisting or concurrent ICI-related thyroiditis have all been linked to ICI-induced autoimmune diabetes (5).

ICI-induced pancreatitis has been reported (12) but to our knowledge, no case series to date characterized the co-existence of pancreatitis and autoimmune diabetes.

## Methods

In this retrospective cohort study, patients with ICI-induced autoimmune diabetes were identified from patients seen at our longitudinal endocrine clinic. We referred to the American Diabetes Association (ADA) guideline (13) for the criteria to make the diagnosis of autoimmune diabetes. We defined patients with ICI-induced autoimmune diabetes as patients without a history of type 1 diabetes who, upon receiving ICI treatment for underlying malignancy, developed insulin-dependent diabetes following initiation of ICI treatment. The study period (January 2014 to June 2020) was defined as the time from the first patient identified with ICI-induced autoimmune diabetes in our institution to June 2020, the time of last chart review. We performed chart reviews to study demographic characteristics, clinical features, biochemical laboratory characteristics, and medical treatment. This study was approved by our institutional IRB.

### Statistical Analyses

Descriptive statistics were calculated for continuous variables. The data were expressed as median with range.

## Results

Eleven patients met criteria for ICI-related diabetes during the study period.

### Demographics and ICI Treatment

ICIs were first administered in this cohort between 2014 and 2017. The onset of autoimmune diabetes was documented between 2014 and 2018. Demographic data are summarized in **Table 1**. Among 11 patients, 7 were female and 4 were male. Primary malignancies included melanoma (n=4), non-small cell lung cancer (n=3), breast cancer (n=2), renal cancer (n=1) and gastric cancer (n=1). The median age of onset of autoimmune diabetes was 61 years (range 36-78). The median body mass index (BMI) was 24 kg/m^2^ (range 19-33). Six patients received pembrolizumab monotherapy, one patient received nivolumab monotherapy, two patients received ipilimumab-nivolumab combination therapy and one patient received ipilimumab-nivolumab combination therapy followed by pembrolizumab. Six of the 11 subjects were deceased at the end of the study period. No subjects in this cohort were exposed to high-dose glucocorticoids at the onset of autoimmune diabetes.

**Table 1:** Demographic data.

Patient	Age	Sex	BMI	Type of cancer	ICI	Alive
1	68	F	21.8	Breast cancer	Pembrolizumab	No
2	72	M	21.8	Non-small cell lung cancer	Nivolumab/Ipilimumab	Yes
3	36	F	31.9	Malignant melanoma	Nivolumab/Ipilimumab	Yes
4	41	F	32.9	Malignant melanoma	Pembrolizumab, Ipilimumab	No
5	61	F	21	Breast cancer	Pembrolizumab	No
6	62	F	23.5	Non-small cell lung cancer	Nivolumab	No
7	60	M	24.6	Renal cell (clear cell) cancer	Pembrolizumab	Yes
8	44	F	19.8	Malignant melanoma	Nivolumab/Ipilimumab, Pembrolizumab	Yes
9	55	F	19	Gastric cancer	Nivolumab/Ipilimumab	No
10	78	M	25.8	Malignant melanoma	Pembrolizumab	No
11	68	M	21.8	Non-small cell lung cancer	Pembrolizumab	Yes

F, Female; M, Male; ICI, Immune checkpoint inhibitors; BMI, Body mass index.

### Clinical Characteristics of Autoimmune Diabetes

Eight patients had a lipase and/or amylase level measured at the onset of hyperglycemia/autoimmune diabetes. Only 2/8 (25%) had elevated lipase and/or amylase (**Table 2**).

**Table 2:** Clinical and biochemical features.

Patients	BG level at onset of DM (mg/dl)	DKA	Other endocrine toxicity	A1C at onset of DM	C-peptide (ng/ml)	Time to onset of DM after ICI (weeks)	GAD65	Amylase (U/L)	Lipase (U/L)
1	1970	Yes	Thyroiditis	9.5	0.3	9	0.28	-	375
2	210	No	Pre-existing hypothyroidism, thyroiditis	9.5	0.7	10	0	53	51
3	243	No	Thyroiditis and hypophysitis	7.8	1	40	43.1	16	22
4	269	No	Thyroiditis and hypophysitis	6.9	0	15	0.02	57	52
5	340	No	none	7.6	0.4	26	0.02	47	37
6	256	Yes	none	7.3	0	11	0.16	–	630
7	222	No	Pre-existing hypothyroidism, thyroiditis	7.6	1.8	6	2.67	–	–
8	386	Yes	Pre-existing hypothyroidism, thyroiditis and hypophysitis	7.4	0.6	122	0	42	27
9	684	Yes	Thyroiditis	7.4	0.3	5	23.8	16	<10
10	768	Yes	none	6.2	0.5	11	0	18	21
11	636	Yes	none	6.2	0.3	10	0.03	–	–

BG, blood glucose; DM, diabetes mellitus; DKA, diabetic ketoacidosis; ICI, immune checkpoint inhibitor; GAD65, glutamic acid decarboxylase 65 antibody. Reference range: GAD65, <=0.02 nmol/L; Amylase, 3-100 U/L; Lipase, 13-60 U/L.

The median hemoglobin A1C at onset of autoimmune diabetes was 7.4% (range 6.2-9.5%). The median onset time from initial ICI dose to onset of ICI-induced autoimmune diabetes was 11 weeks (range 5-122 weeks). The median glucose level at presentation was 443 mg/dl (range 210-1970 mg/dl). All patients had measurement of C-peptide and concomitant blood glucose values. The median C-peptide value measured with concomitant plasma glucose >210 mg/dl at the time of presentation of diabetes was low at 0.4 ng/ml (range 0-1.8). Six patients (54.5%) presented with diabetic ketoacidosis (DKA). Seven patients (64%) developed ICI-induced thyroiditis, including 27% who had preexisting hypothyroidism at the time of onset of autoimmune diabetes and 36% who were newly diagnosed with thyroiditis at the time of onset of autoimmune diabetes. Three patients (27%) had coexistence of hypophysitis, thyroiditis and autoimmune diabetes.

Approximately half had positive GAD65 antibody (54% or 6/11). Other tested antibodies including islet antigen 2 (IA-2), islet cell antibody (ICA) and zinc transporter 8 antibody (ZNT8) were negative. (Data not shown).

## Discussion

In this retrospective cohort study, we found 8 and 6 patients had concomitant lipase and amylase tests at the presentation of autoimmune diabetes respectively. 25% (2/8) had an increased lipase level, and no patient (0/6) had an increased amylase level. Interestingly, although ICI-induced hypophysitis can lead to isolated pituitary hormone deficiencies such as isolated ACTH deficiency (14, 15), to our knowledge, no case series has been published analyzing ICI-induced selective pancreatic endocrine (islet) and exocrine toxicity. This is the first retrospective cohort demonstrating a selective islet toxicity in most of the patients who developed ICI-induced autoimmune diabetes. In addition, other patients (n = 4) in our longitudinal clinic developed pancreatitis with elevated lipase but did not develop autoimmune diabetes, suggestive of a selective exocrine pancreas toxicity. The findings suggest ICI can induce selective pancreatic endocrine or exocrine toxicity. Because simultaneous cross-sectional abdominal imaging studies were not routinely done in these cases, we could not analyze coexistence of radiographic evidence of pancreatitis. Notably, the incidence of ICI-induced pancreatitis was only 2.7% in a recent meta-analysis study (16). The incidence of 25% of pancreatitis in our cohort suggests an increased risk of pancreatitis in patients who develop ICI-induced autoimmune diabetes (17). We performed literature search to identify the correlation between pancreatic enzyme and ICI-induced autoimmune diabetes, there were only 2 case reports had lipase value and both were elevated (10, 18). This find underscores the novelty of this study to evaluate ICI-induced pancreatic toxicity.

In our analysis, all cases of ICI-induced autoimmune diabetes were associated with either anti-PD-1 monotherapy or combination anti-PD-1/anti-CTLA-4 therapy; no cases were associated with anti-CTLA-4 monotherapy (**Table 1**). This is consistent with previous studies (5, 11, 19). The majority of the patients (8/11) in this study had normal BMI at the onset of autoimmune diabetes. The median age of onset of ICI-induced autoimmune diabetes was 61 years. The onset age of diabetes probably reflects the age of development of malignancies and age at ICI treatment, although it is worth noting that ICI-induced autoimmune diabetes has been reported in a 12-year-old patient with relapsed classical Hodgkin lymphoma (20).

A strong link between thyroid autoimmunity and spontaneous type 1 diabetes has been well-documented (21, 22). Similarly, the coexistence of ICI-induced thyroiditis and autoimmune diabetes has been described (17, 23). In our study, 27% of patients (3/11) had preexisting hypothyroidism, and ICI-induced thyroiditis manifested as transient thyrotoxicosis while these patients were on a steady dose of levothyroxine replacement. It is unclear if preexisting thyroid autoimmunity predisposed patients to develop ICI-induced autoimmune diabetes. We identified 4 patients (36%) with normal thyroid function prior to initiation with ICIs who developed ICI-induced thyroiditis. While a link between these conditions is suspected in the setting of ICI exposure, whether they have a shared underlying mechanism remains to be determined.

We also identified the coexistence of hypophysitis and thyroiditis in 3 patients (27%). This occurred following different regimens: one subject received combination anti-PD1 and anti-CTLA-4 therapy, one received combination anti-PD-1 and anti-CTLA-4 followed by anti-PD-1 monotherapy (**Table 1**), and one subject received anti-PD-1 monotherapy. These findings suggest that varied mechanisms might have mediated autoimmunity in endocrine organs.

About half (54%) of our subjects had positive GAD65 antibodies. This is similar to but slightly higher than previous reports in which GAD65 antibodies were positive in 40% of patients (23) but different from spontaneous type 1 diabetes in which over 95% of patients have developed at least one positive autoantibody by the time of diagnosis (24). In addition to GAD65 antibody, other autoantibodies related to type 1 diabetes were reported positive in some patients with ICI-induced autoimmune diabetes, albeit at a lower rate (23). In a recent case report with systemic review by online literature search, 88 patients with ICI-induced autoimmune diabetes, there were 47 (53%) patients had positive pancreatic antibodies. Among the antibodies, there were 43 patients had positive GAD antibody, 10 patients had positive IA-2 antibody, 9 patients had positive insulin antibody, 3 patient had positive ICA antibody and 1 patient had positive ZnT8 antibody (10). In our cohort, no patients had positive titers of anti-islet cell antibody or anti-insulin antibody. GAD65 antibody therefore appears to be the most sensitive autoantibody related to ICI-induced autoimmune diabetes. Notably, as about half of patients had a negative GAD65 antibody, GAD65 antibody positivity should not be used as a required criterion to make the diagnosis of ICI-induced autoimmune diabetes.

Blood glucose at initial presentation of autoimmune diabetes was markedly elevated at 443 mg/dl (range 210-1970 mg/dl) (**Table 2**) in the setting of low C-peptide level (median 0.4 ng/ml, range 0-1.8 ng/ml), which is consistent with previous findings (23) and suggests rapid loss of beta-cell function in ICI-induced autoimmune diabetes. Although the median glucose level was above 400 mg/dl, the median A1C was 7.4%. The discordance of blood glucose and A1C at presentation of autoimmune diabetes again indicates rapid loss of beta-cell function. As A1C reflects the average blood glucose level over the past 120 days through percentage of glycated hemoglobin, acute onset of severe hyperglycemia within the prior days to weeks would not be expected to significantly impact the A1C. Early identification of ICI-induced autoimmune diabetes is critical because timely initiation with insulin treatment can potentially avoid severe complications from autoimmune diabetes, such as DKA. It is therefore important to educate all patients treated with ICI regarding symptoms related to hyperglycemia of which to be aware.

Management of ICI-induced autoimmune diabetes is changeling. Many patients required insulin pump and continuous glucose monitoring (CGM). Given the nature of type 1 diabetes, the insulin doses overall were low and stable. The patient had no signs of insulin resistance.

Limitations of this study include its retrospective design and small sample size. Future prospective trials and larger sample sizes are needed to better characterize ICI-induced autoimmune diabetes.

## Data Availability Statement

The raw data supporting the conclusions of this article will be made available by the authors, without undue reservation.

## Ethics Statement

The studies involving human participants were reviewed and approved by Brigham and Women’s Hospital Institutional Review Board. Written informed consent for participation was not required for this study in accordance with the national legislation and the institutional requirements.

## Author Contributions

Conception and study design: LM. Development of methodology: LM and AZ. Acquisition of data from chart review and analysis: AZ and FW. Wrote and edited the manuscript: LM and AZ. Review and revision of manuscript: LM, AZ, FW, L-SC, and MM. All authors contributed to the article and approved the submitted version.

## Conflict of Interest

The authors declare that the research was conducted in the absence of any commercial or financial relationships that could be construed as a potential conflict of interest.
